# Acupuncture for Hypertension in Animal Models: A Systematic Review and Meta-Analysis

**DOI:** 10.1155/2021/8171636

**Published:** 2021-10-11

**Authors:** Ling-Yong Xiao, Zheng Li, Yu-Zheng Du, Hui-Yan Shi, Si-Qi Yang, Yue-Xin Zhang, Rui-Yu Li, Wan-Ling Lin, He-Yang Wang, Xiao-Yu Dai

**Affiliations:** ^1^Clinical Department of Acupuncture and Moxibustion, First Teaching Hospital of Tianjin University of Traditional Chinese Medicine, Tianjin 300193, China; ^2^National Clinical Research Center for Chinese Medicine Acupuncture and Moxibustion, Tianjin 300193, China; ^3^Affiliated Zhongshan Hospital of Dalian University, Dalian 116001, China

## Abstract

**Objective:**

The aim of this study was to summarize and evaluate the efficacy of acupuncture in hypertension animal study.

**Methods:**

Studies were searched from six databases, including Medline, Embase, Chinese National Knowledge Infrastructure, Wanfang Data, VIP information database, and Chinese Biomedical Literature Database. Study quality of each included study was evaluated according to the Animal Research: Reporting of In Vivo Experiments (ARRIVE) guidelines, and the risk of bias was evaluated by the Systematic Review Centre for Laboratory Animal Experimentation (SYRCLE) tool. Systolic blood pressure (SBP), diastolic blood pressure (DBP), and mean arterial pressure (MAP) were selected as outcomes. Meta-analyses were performed using Stata 12.0 software. The effect size was calculated by combining SBP/DBP/MAP data with the random effects model, respectively.

**Results:**

67 studies containing 1522 animals were included. According to the ARRIVE guideline, 8 items were assessed as poor and 4 items were assessed as excellent. According to the SYRCLE tool, all studies were judged as having high risk of bias. Compared with the hypertension group, the pooled results showed significant antihypertension effects of acupuncture for SBP, DBP, and MAP. Similarly, compared with the sham-acupuncture group, the pooled results showed significant antihypertension effects of acupuncture for SBP, DBP, and MAP.

**Conclusion:**

Although pooled data suggested that the acupuncture group was superior to the hypertension group or sham-acupuncture group for SBP/DBP/MAP, the presentation of poor methodological quality, high risk of bias, and heterogeneity deserves cautious interpretation of the results.

## 1. Introduction

Hypertension is a major modifiable risk factor for cardiovascular and cerebral vascular disease [1, 2], affecting about 1.39 billion people worldwide [3]. The main treatment modality for hypertension is pharmacological treatment. Although considerable progress has been made in the field of antihypertensive medicines, only 13.8% of adults with hypertension had their blood pressure (BP) controlled up to the standard worldwide [3]. Because of its relative safety, acupuncture has led to a growing interest among nonpharmacological complementary therapies, in the treatment of hypertension. Also, it has been shown potential in lowering BP, although the effects were not proven sustaining [4].

Plenty of research studies focusing on antihypertensive mechanisms of acupuncture have shown that acupuncture elicits antihypertensive effects through the regulation of renin-angiotensin-aldosterone system, vascular endothelium function, oxidative stress, neuroendocrine system, and so on [5]. However, the efficacy and mechanisms of acupuncture for experimentally induced hypertension have not been systematically investigated yet. The scientific theory for systematic reviews of animal studies was initially summarized in a commentary published in The Lancet [6]. A systematic review of preclinical animal studies contributes to translational medicine and potentially brings about more precise medical care decisions [7]. In addition, systematic review of preclinical evidence could inform the design and contribute to success of future clinical studies, indicate the necessity of further research, reduce unnecessary study replication, and implement the principle of “replacement, refinement, and reduction of animals” in animal research [8]. Thus, the aim of present review is to synthesize and appraise pooling results of acupuncture's antihypertensive effects in animal models, quantitively assess influencing factors of acupuncture's efficacy, explore future study direction, provide clues for clinical studies, and assess potential publication bias and its influence.

## 2. Materials and Methods

### 2.1. Search Strategy

The following sources were searched from inception to July 2020: Medline, Embase, Chinese National Knowledge Infrastructure (CNKI), Wanfang Data, VIP information database, and Chinese Biomedical Literature Database. In an effort to identify further published and unpublished research studies, we retrieved the reference lists of relevant reports and review articles identified from electronic databases. Following terms combined were used for searching: acupuncture OR acupoint, hypertension OR blood pressure, animal OR pre-clinical study OR mechanism study.

### 2.2. Inclusion Criteria

Studies were included if all of the following standards were met: sustaining high blood pressure (HBP) animal model, baseline systolic blood pressure (SBP) ≥ 140 mmHg, diastolic blood pressure (DBP) ≥ 90 mmHg [9, 10]; at least one of the following was used as outcome measures: SBP, DBP, and MAP; and SBP/DBP/MAP was compared with those of hypertension animals receiving sham-acupuncture or no treatment.

### 2.3. Exclusion Criteria

Studies were excluded if any of the following standards was met: the HBP was induced by stress or adrenaline administration and was not lasting; studies were conducted to compare the effects of different acupuncture methods on hypertension, with no treatment or sham-acupuncture treatment as control; studies did not use BP values as outcomes; and duplicate publications.

### 2.4. Selection of Studies

**Figure 1:**
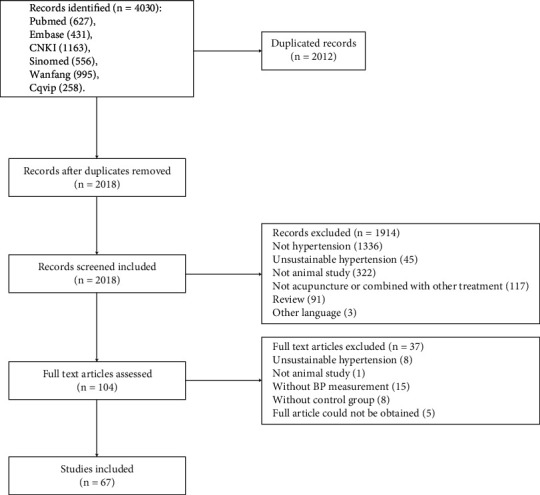
Flowchart of the study selection.

Two reviewers screened the titles and/or abstracts of searched studies and excluded obviously irrelevant studies, such as clinical studies, reviews, and nonhypertension and nonacupuncture studies. The full texts were obtained for the remaining studies. A flowchart of study selection is presented in Figure 1.

### 2.5. Data Extraction

Two reviewers independently assessed the eligible studies and extracted data using a predefined template. The following items were extracted: publication information, experimental animal information, type of animal model, acupuncture manipulation methods, outcome measurements, data of mean outcome, standard deviation, and sample of animals in the acupuncture as well as the control groups. If the results were only shown by chart, the data were obtained by accurate measurement from the figures by software Digxy1.0.0.1. The missing information was sought by sending e-mail or telephone call to the corresponding author of the article. Stata 12.0 software was used for data analysis.

### 2.6. Quality Assessment

The methodological quality of the included studies was assessed according to the Animal Research: Reporting of In Vivo Experiments (ARRIVE) guidelines, the standards of reporting in animal research. 20 items were included in the guideline. Standards of grading were adjusted based on previous report [11, 12]. Items “1” and “11” were marked a lowest score of 0 and a highest score of 1 (0 = inaccurate, not concise, or not reported; 1 = accurate, concise, or reported). The other items (2, 3, 4, 5, 6, 7, 8, 9, 10, 12, 13, 14, 15, 16, 17, 18, 19, and 20) were marked a lowest score of 0 and a highest score of 2 (0 = clearly inaccurate or not reported; 1 = possibly accurate, unclear, or incomplete; 2 = clearly accurate). The total scores of one study ranged from 0 to 38. The category score was sum of scores by each item. The maximum score was the maximum possible score, i.e., when each item was assessed as clearly accurate [11]. We calculated a ratio quality score/maximum score, generating three possible quality intervals of which 0.8–1 was considered “excellent,” 0.5–0.8 was considered “average,” and scores below 0.5 was considered “poor.”

### 2.7. Bias Assessment

The risk of bias was evaluated with the Systematic Review Centre for Laboratory Animal Experimentation (SYRCLE) tool [13]. This tool contains 10 items about the selection, performance, detection, attrition, reporting bias, etc. Each item was labeled yes, no, or unclear to score selected articles. Studies with 1 item recognized to be high risk of bias were considered to have an entire high risk of bias. Studies with unclear risk of bias for at least one item were considered to be at unclear risk of bias, and studies with low risk of bias in all items were assessed as low risk of bias [14].

The assessment of the methodological quality and risk of bias was performed by two authors separately. Any divergence was resolved through discussion with a third reviewer.

### 2.8. Statistical Analysis

Meta-analyses were performed using Stata 12.0 software. Outcome measures of SBP, DBP, and MAP were considered as continuous data. The effect size was calculated by combining these data with the random effects model, respectively. Publication bias was assessed with a funnel plot and Egger's test. The *I*^2^ statistic was used for detection of heterogeneity. If the *I*^2^ statistic was higher than 50%, we considered significant heterogeneity was present. To explore the impact of factors potentially influencing the BP outcome, subgroup analyses were conducted for the following factors: acupuncture methods used, age of acupuncture initiation, age of BP measurement, duration of each acupuncture session, total acupuncture sessions, and acupuncture treatment frequency. Sensitivity analyses were performed by deleting one study at a time from the pooled studies.

## 3. Results

According to search criteria, we retrieved 4030 potentially relevant records from 6 databases, of which 2012 were duplicate records. After screening titles and abstracts of remaining 2018 records, 1914 records were excluded for one or more of the following reasons: (1) not research studies of hypertension or hypertension combined with other conditions, (2) unsustainable hypertension, e.g., the HBP was caused by cold or electric stress, (3) not animal study, (4) not an acupuncture study or acupuncture combined with other therapy, (5) review articles, and (6) other language. By browsing the whole text of the remaining 104 records, a total of 37 studies were excluded for one or more of the following reasons: (1) unsustainable hypertension, (2) not animal study, (3) without BP measurement, (4) without control group, and (5) full article could not be obtained. Finally, 67 eligible studies were included, and 1522 animals were involved in the meta-analysis.

### 3.1. Study Characteristics

Totally, 1522 animals were included for comparison. Of the hypertension models in these studies, 63 used SHR, 1 used AngII infusion [44], and 3 used renal artery stenosis [17, 21, 22]. 3 studies simultaneously assessed SBP/DBP/MAP as the outcome measures [42, 48, 52]. 20 studies assessed SBP and DBP as the outcome measures [23, 32, 35, 36, 38, 41, 43, 45, 47, 49, 50, 53, 55, 56, 60, 62, 68, 72, 79, 80]. 3 studies assessed SBP and MAP as the outcome measures [37, 58, 70]. 35 studies assessed only SBP as the outcome measures [9, 17–22, 24–27, 29–31, 33, 34, 39, 40, 46, 51, 54, 57, 59, 61, 63–65, 67, 69, 71, 73, 75–78]. 6 studies assessed only MAP as the outcome measure [15, 16, 28, 44, 66, 74]. 40 studies adopted manual acupuncture [9, 17, 20, 25, 29, 32–41, 43, 45–47, 50, 51, 53, 54, 57–63, 65–68, 70, 71, 75–78], of which 35 adopted manipulation instead of simple needle penetration [9, 17, 20, 25, 29, 32–41, 43, 45–47, 50, 51, 53, 54, 57, 59–61, 63, 65, 66, 68, 71, 76–78]. 20 studies used EA as intervention [15, 16, 18, 21–23, 27, 28, 30, 31, 42, 44, 48, 52, 64, 69, 72–74, 79]. 4 studies combined manipulation and EA as intervention [24, 49, 55, 56]. 2 studies used other auxiliary devices combined with acupuncture as treatment [19, 80]. The needle retaining time ranged from 0.5 minute to 30 minutes. The duration of acupuncture ranged from 1 time to 48 times. Animal age of treatment initiation ranged from 8 weeks to 34 weeks. Study characteristics are shown in Table 1.

### 3.2. Quality Assessment

Twenty items were evaluated according to the ARRIVE guidelines. According to ratio quality score/maximum score, the items 5/6/10/12/13/14/17/18 were considered to be poor. The items 2/3/4/7/9/11/19/20 were considered to be average. Also, the items 1/8/15/16 were considered to be excellent. Detailed information of identified studies' quality is shown in Table 2.

### 3.3. Risk of Bias

Risk of bias was appraised for each included study. 37 studies were thought to have low risk of sequence generation. 23 studies were judged as having low risk of baseline characteristics. No study was considered to have low risk of allocation concealment, random housing, blinding against performance bias, random outcome assessment, and blinding against detection bias. 56 studies were thought to have low risk of incomplete data. All of the 67 studies were thought to have low risk of selective outcome reporting and other bias. Thus, all studies were judged as having high risk of bias. Details of bias assessment information of identified studies are shown in Table 3.

### 3.4. Effectiveness

#### 3.4.1. SBP

**Figure 2:**
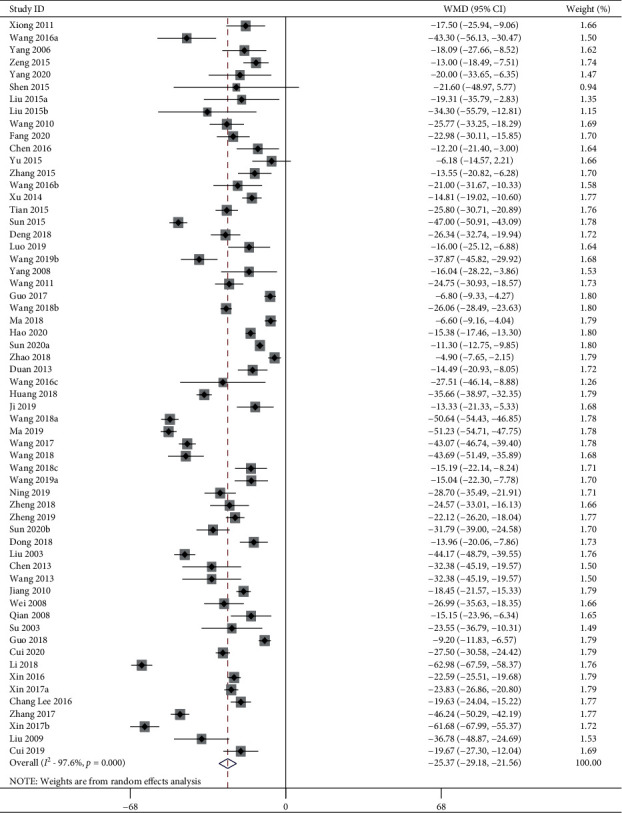
SBP forest plots: acupuncture vs. hypertension. WMD, weighted mean difference.

**Figure 3:**
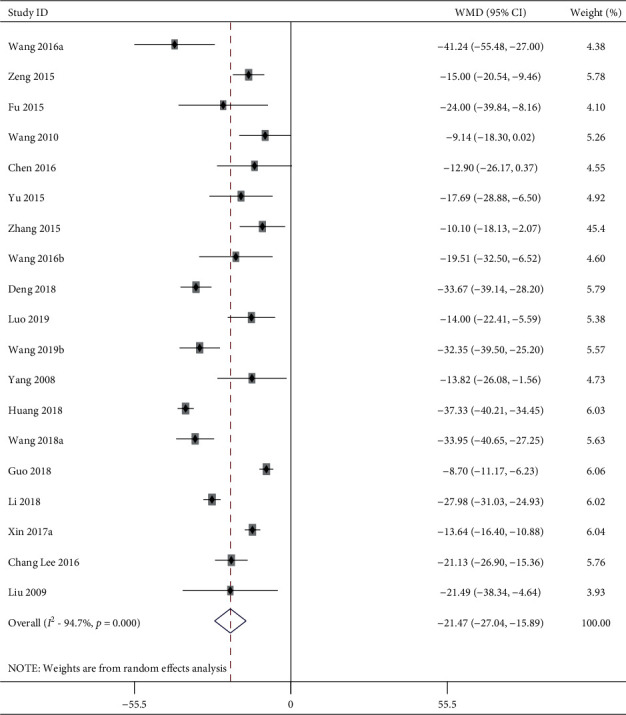
SBP forest plots: acupuncture vs. sham-acupuncture. WMD, weighted mean difference.

61 studies including 1385 animals conducted the meta-analysis of SBP value. Compared with the hypertension model group, acupuncture showed a mean reduction of SBP for 25.37 mmHg with significant heterogeneity in a pooled analysis of 60 studies (Figure 2) (MD −25.37, 95% CI: −29.18 to −21.56, *P* < 0.0001; heterogeneity: chi^2^ = 2746.83, d*f* = 59 (*P* < 0.0001), *I*^2^ = 97.6%). Compared with the sham-acupuncture group, acupuncture showed a mean reduction of SBP for 21.47 mmHg with statistically significant heterogeneity in a pooled analysis of 19 studies (Figure 3) (MD −21.47, 95% CI: −27.04 to −15.89, *P* < 0.0001; heterogeneity: chi^2^ = 342.60, d*f* = 18 (*P* < 0.0001), *I*^2^ = 94.7%).

#### 3.4.2. DBP

**Figure 4:**
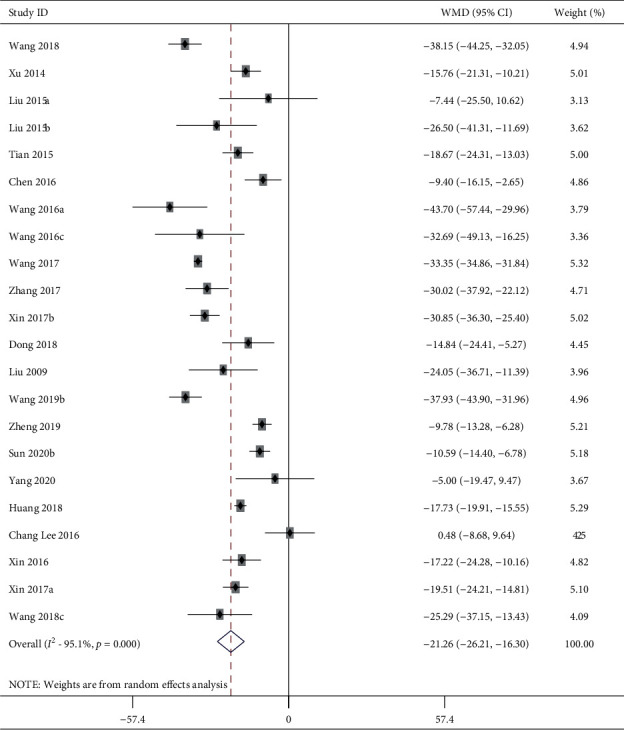
DBP forest plots: acupuncture vs. hypertension. WMD, weighted mean difference.

**Figure 5:**
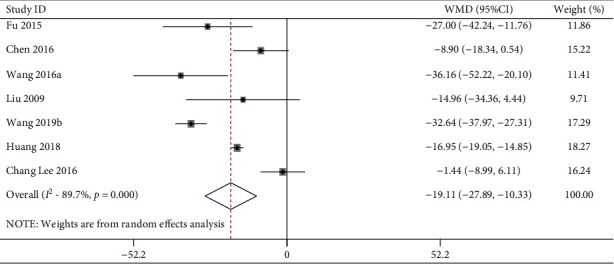
DBP forest plots: acupuncture vs. sham-acupuncture. WMD, weighted mean difference.

23 studies including 523 animals conducted the meta-analysis of DBP value. Compared with the hypertension model group, acupuncture showed a mean reduction of DBP for 21.26 mmHg with statistically significant heterogeneity in a pooled analysis of 22 studies (Figure 4) (MD −21.26, 95% CI: −26.21 to −16.30, *P* < 0.0001; heterogeneity: chi^2^ = 425.69, d*f* = 21 (*P* < 0.0001), *I*^2^ = 95.1%). Compared with the sham-acupuncture group, acupuncture showed a mean reduction in DBP for 19.11 mmHg with statistically significant heterogeneity in a pooled analysis of 7 studies (Figure 5) (MD −19.11, 95% CI: −27.89 to −10.33, *P* < 0.0001; heterogeneity: chi^2^ = 58.32, d*f* = 6 (*P* < 0.0001), *I*^2^ = 89.7%).

#### 3.4.3. MAP

**Figure 6:**
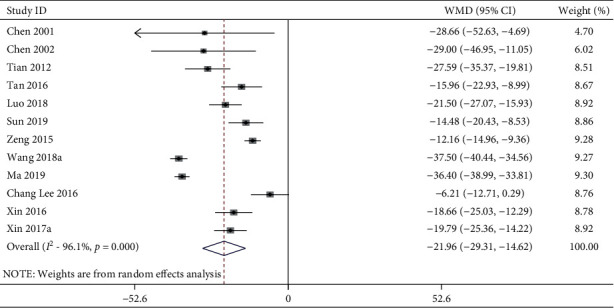
MAP forest plots: acupuncture vs. hypertension. WMD, weighted mean difference.

**Figure 7:**
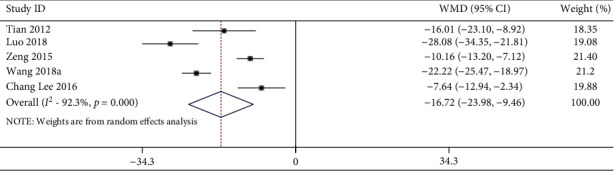
MAP forest plots: acupuncture vs. sham-acupuncture. WMD, weighted mean difference.

12 studies including 274 animals conducted the meta-analysis of MAP value. Compared with the hypertension model group, acupuncture showed a mean reduction of MAP for 21.96 mmHg with statistically significant heterogeneity in a pooled analysis of 12 studies (Figure 6) (MD −21.96, 95% CI: −29.31 to −14.62, *P* < 0.001; heterogeneity: chi^2^ = 284.15, d*f* = 11 (*P* < 0.0001), *I*^2^ = 96.1%). Compared with the sham-acupuncture group, acupuncture showed a mean reduction in MAP for 16.72 mmHg with statistically significant heterogeneity in a pooled analysis of 5 studies (Figure 7) (MD −16.72, 95% CI: −23.98 to −9.46, *P* < 0.0001; heterogeneity: chi^2^ = 51.97, d*f* = 4 (*P* < 0.0001), *I*^2^ = 92.3%).

### 3.5. Subgroup Analysis and Sensitivity Analysis

To investigate potential factors which influenced the BP measures, we stratified the included studies according to factors, as shown in Tables S1–S5. The subgroup analysis of MAP comparing acupuncture and sham-acupuncture was not conducted due to limited number of studies.

For SBP/DBP/MAP, sensitivity analyses showed that the results did not largely change after omitting any one study (Figures S1–S6).

### 3.6. Assessment of Publication Bias

**Figure 8:**
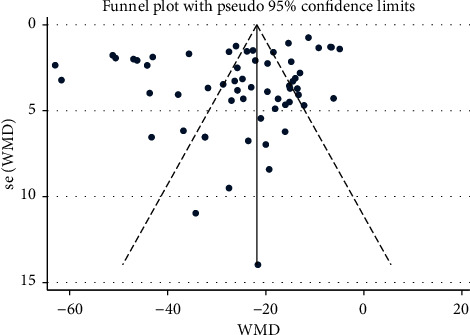
Funnel plot of SBP: acupuncture vs. hypertension.

**Figure 9:**
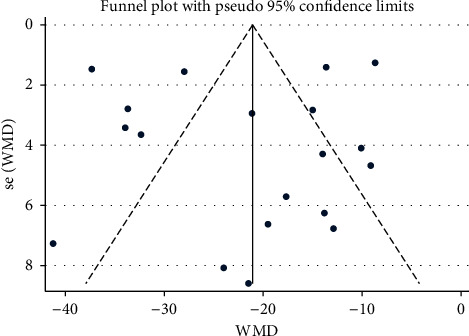
Funnel plot of SBP: acupuncture vs. sham-acupuncture.

**Figure 10:**
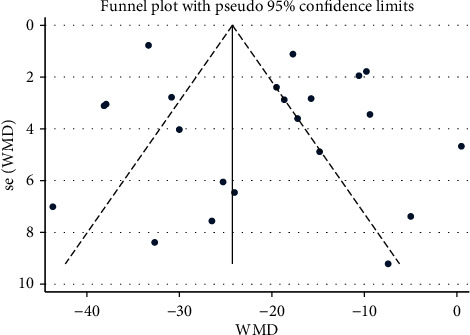
Funnel plot of DBP: acupuncture vs. hypertension.

**Figure 11:**
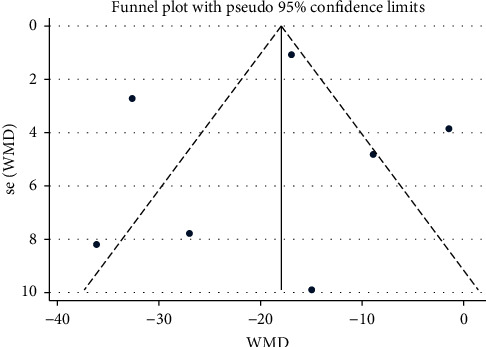
Funnel plot of DBP: acupuncture vs. sham-acupuncture.

**Figure 12:**
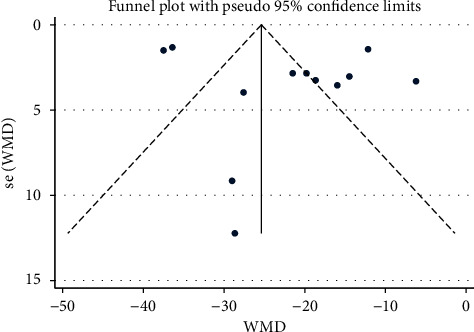
Funnel plot of MAP: acupuncture vs. hypertension.

**Figure 13:**
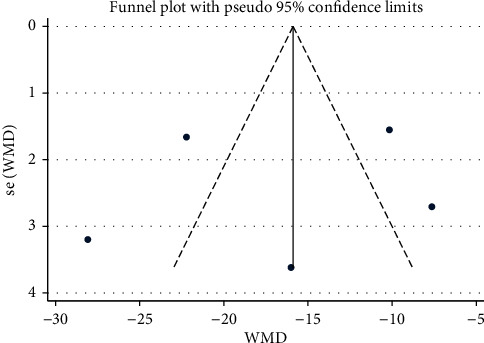
Funnel plot of MAP: acupuncture vs. sham-acupuncture.

Funnel plot of all outcome measures between different groups (Figures 8–13) showed symmetry, indicating no significant publication bias. Egger's test of different comparisons of SBP/DBP/MAP showed no publication bias present. Details of Egger's test are shown in Table S6.

### 3.7. Related Mechanisms

The underlying molecular mechanisms of acupuncture involved in the present review for hypertension are summarized as follows: (1) inhibition of inflammatory factors including tumor necrosis factor-*α* (TNF-*α*) [21], interleukin-6, and C-reactive protein [40, 46], as well as inflammation-related genes [63], through toll-like receptor 4 [66] in the PVN; (2) reduction of oxidative stress reactions [64, 72–74, 79] by inhibiting nicotinamide adenine dinucleotide phosphate (NADPH) oxidase activity by downregulation of p38 mitogen-activated protein kinases and extracellular signal-regulated protein kinase 1/2 [58]; (3) inhibition of AngII activity in the plasma [23, 32, 62, 67, 75], myocardium [59, 71], thoracic aorta [69], rostral ventrolateral medulla [62], kidney [62], etc. and reversion of the artery remodeling [53] and left ventricle [45, 57] through mitogen-activated protein kinase signaling [24]; (4) inhibition of glucocorticoid system, including corticotropin-releasing hormone, adrenocorticotropic hormone, and cortisol and glucocorticoids receptor [48, 52]; and (5) mediation of the balance between vasorelaxation factors and vasomotor factors and improvement of the endothelial dysfunction [15, 16, 21, 25–27, 34, 39, 49, 57, 59]. This is consistent with the genomic study of acupuncture, which suggested acupuncture may regulate multiple biological processes mainly involving oxidative stress, inflammation, and vascular endothelial function [70].

## 4. Discussion

To our knowledge, we present the first systematic review and meta-analysis of the efficacy of acupuncture in treating animal models of hypertension. For the outcome measures, including SBP/DBP/MAP, our meta-analysis showed better results in the acupuncture group than in the model or sham-acupuncture group. This indicates that acupuncture might potentially decrease HBP, though there was high heterogeneity among the results of the analysed studies.

### 4.1. Limitations

Limitations are enumerated as follows. First, being limited in English and Chinese databases, other language studies may be missed. The absence of other language studies may bring about selective bias. Second, negative results were hardly published. Thus, the whole body of positive results might bring about exaggerated efficacy. Third, most included studies are defective in terms of randomization, allocation concealment, blinding assessment, and sample size calculation by both assessment scales, which are central for the preclinical study design criteria [81]. Low methodological quality and high risk of bias weakened the robustness of the current preclinical evidence. Fourth, the high level of heterogeneity among different studies implied not only differences of laboratory animals but also the acupuncture method details. Though subgroup analyses according to several factors were conducted to explore possible sources of heterogeneity, the results failed to completely account for the heterogeneity. Thus, the present study should be interpreted cautiously.

### 4.2. Efficacy Assessment

Although the effectiveness of acupuncture's antihypertension in most studies was demonstrated by the value of BP, the ultimate goal of hypertension treatment is to prevent target organ damage and decrease mortality. Among the 67 included studies, 15 assessed the target organ damage, including left ventricle, kidney, and blood vessel remodeling. Limited by study period, none of the studies assessed the mortality of hypertension animals. Thus, target organ damage should be paid attention to in future acupuncture antihypertension studies.

Although blood pressure variability (BPV) is not a physiological index daily detected in clinical practice, it is deemed as a prognostic factor independent of BP value for cardiovascular mortality [82, 83]. Evidence shows that cardiac accident of hypertension may be the consequence of elevated variability but not BP alone [84, 85]. In addition, there is a strong correlation between elevated BPV and aggravated target organ damage [86]. Only 1 study assessed the effect of acupuncture on BPV [58]. Analysis of BPV may provide information about autonomic function, and there are also studies which demonstrated that acupuncture has the potential to mediate autonomic function [87]. Thus, exploring the effect of acupuncture on hypertension animal BPV may contribute to finding out the potential efficacy of acupuncture.

### 4.3. Implications

Preclinical studies usually provide evidence basis for clinical studies. To demonstrate the scientific function pathway of acupuncture, animal studies should be designed, study data should be analysed, and the result should be reported appropriately and transparently. There is a wealth of evidence showing the poor design of animal research [88], which is an obstacle to advance animal research into promising achievement for human disease. The ARRIVE guidelines have been issued since 2010 to unify the criteria of reporting in animal research and were utilized by this review to assess the quality of paper in respect of acupuncture's antihypertension effects. The ARRIVE [89] is a reporting guideline containing a 20-entry checklist about each part of publishing papers. The SYRCLE tool was applied to assess the internal validity and risk of bias within individual studies [13]. In addition to items of ARRIVE and SYRCLE, acupuncture's details including acupuncture manipulation, treatment duration, if electroacupuncture is used, the frequency and intensity of pulse method, etc. should be especially noted. Although these assessment tools could be used in acupuncture preclinical study [90], the need for adjusted and unified criteria in reporting of acupuncture preclinical study is imperative. Similar to the Standards for Reporting Interventions in Clinical Trials of Acupuncture (STRICTA) [91], a specialized guideline for acupuncture's preclinical study should be formulated in order to promote study's transparency and repeatability.

## 5. Conclusion

To our knowledge, we present the first systematic review and meta-analysis of the efficacy of acupuncture in treating animal models of hypertension. Although pooled data suggested that the acupuncture group was superior to the hypertension group or sham-acupuncture group for SBP/DBP/MAP, the presentation of poor methodological quality, high risk of bias, and heterogeneity deserves cautious interpretation of the results.

## Figures and Tables

**Table 1:** Characteristics of included studies.

Study	Animals (species, sex, weight)	Model	Control group	Outcome measurement	Week-age of acupuncture initiation (weeks)	Week-age of BP measurement (weeks)	Acupuncture methods	Acupoints	Acupuncture retaining time (minutes)	Acupuncture duration (sessions)
Chen 2001 [15]	SHR, male and female, 189.17 ± 31.26 g	SHR	HTN	MAP	10	13	EA	ST36	15	12
Chen 2002 [16]	SHR, male and female, NR	SHR	HTN	MAP	11	14	EA	ST36	15	15
Liu 2003 [17]	Wistar, NR, 200 ± 20 g	Renal ischemia	HTN	SBP	22	24	Manip	ST36, SP3, PC6, KI1	20	14
Su 2003 [18]	SHR, male, NR	SHR	HTN	SBP	9	22	EA	ST36, LI11	30	45
Yang 2006 [19]	SHR, male, 270 ± 10 g	SHR	HTN	SBP	12	15	Other device	ST36, LR3	3	15
Yang 2008 [20]	SHR, male, 190 ± 10 g	SHR	HTN, Sham-acu	SBP	NR	NR	Manip	LR3	5	7
Wei 2008 [21]	SD, male and female, 190 ± 10 g	Renal ischemia	HTN	SBP	NR	NR	EA	LI11	20	28
Qian 2008 [22]	SD, male and female, 175 ± 25 g	Renal ischemia	HTN	SBP	NR	NR	EA	LI11	20	56
Liu 2009 [23]	SHR, male, 220 ± 20 g	SHR	HTN, Sham-acu	SBP, DBP	NR	NR	EA	HT7	30	1
Jiang 2010 [24]	SHR, male, 150 ± 50 g	SHR	HTN	SBP	34	37	EA	LI11, GB20, SP6	15	18
Wang 2010 [25]	SHR, male, 200 ± 20 g	SHR	HTN	SBP	9	10	Manip	LR3	5	7
Xiong 2011 [26]	SHR, male, NR	SHR	HTN	SBP	13	21	EA	ST36	NR	40
Wang 2011 [27]	SHR, male, 200 ± 20 g	SHR	HTN	SBP	NR	NR	EA	LR3	5	7
Tian 2012 [28]	SHR, male, 220 ± 30	SHR	HTN	MAP	8	16	EA	ST36, DU20	20	28
Duan 2013 [29]	SHR, male, 200 ± 20 g	SHR	HTN	SBP	11	13	Manip	LR3	20	14
Chen 2013 [30]	SHR, male, 385.22 ± 21.26	SHR	HTN	SBP	24	30	EA	ST36, LI11	20	42
Wang 2013 [31]	SHR, male, 360 ± 20 g	SHR	HTN	SBP	26	32	EA	ST36, LI11	20	42
Xu 2014 [32]	SHR, male, 260 ± 20	SHR	HTN	SBP, DBP	12	16	Manip	LR3	10	24
Yu 2014 [33]	SHR, male, 210 ± 10	SHR	HTN, Sham-acu	SBP	12	13	Manip	LR3, KI3	5	7
Shen 2015 [34]	SHR, male, 245 ± 25	SHR	HTN	SBP	17	21	Manip	ST9	1	20
Liu 2015 [35]	SHR, male, 215 ± 25	SHR	HTN	SBP, DBP	17	21	Manip	ST9	1	20
Liu 2015 [36]	SHR, male, 215 ± 25	SHR	HTN	SBP, DBP	17	21	Manip	ST9	1	20
Zeng 2015 [37]	SHR, male, 255 ± 5	SHR	HTN	SBP, MAP	13	15	Manip	LR3	0.5	12
Tian 2015 [38]	SHR, male, 225 ± 25	SHR	HTN	SBP, DBP	12	16	Manip	LR3	10	24
Sun 2015 [39]	SHR, male, 225 ± 25	SHR	HTN	SBP	12	16	Manip	LR3	10	24
Zhang 2015 [40]	SHR, male, 210 ± 10	SHR	HTN, Sham-acu	SBP	12	13	Manip	KI3	5	7
Fu 2015 [41]	SHR, male, 300 ± 10 g	SHR	Sham-acu	SBP, DBP	18	22	Manip	ST9	1	28
Chang Lee 2016 [42]	SHR, male, NR	SHR	HTN, Sham-acu	SBP	NR	NR	EA	LR3	30	21
Chen 2016 [43]	SHR, male, 190 ± 10 g	SHR	HTN, Sham-acu	SBP, DBP	10	11	Manip	LR3	5	7
Tan 2016 [44]	SD, male, 190 ± 10 g	AngII-induced	HTN	MAP	NR	NR	EA	LI11	20	14
Wang 2016 [45]	SHR, male, 250 ± 20 g	SHR	HTN, Sham-acu	SBP, DBP	11	14	Manip	ST9	2	21
Wang 2016 [46]	SHR, male, 210 ± 10	SHR	HTN, Sham-acu	SBP	12	13	Manip	KI3	5	7
Wang 2016 [47]	SHR, male, 250 ± 20	SHR	HTN	SBP, DBP	11	15	Manip	ST36, LR3	20	28
Xin 2016 [48]	SHR, male, 255 ± 15	SHR	HTN	SBP, DBP, MAP	12	20	EA	PC6	30	56
Wang 2017 [49]	SHR, male, 260 ± 9.04	SHR	HTN	SBP, DBP, MAP	12	16	Manip + EA	ST36, LI11	16	20
Zhang 2017 [50]	SHR, male, 190 ± 10 g	SHR	HTN	SBP, DBP	NR	NR	Manip	LR3, PC6	30	15
Guo 2017 [51]	SHR, male, 190 ± 10 g	SHR	HTN	SBP	9	13	Manip	ST9	20	28
Xin 2017 [52]	SHR, male, 255 ± 15 g	SHR	HTN	SBP, DBP, MAP	13	21	EA	PC6	30	56
Xin 2017 [53]	SHR, male and female, 220 ± 20 g	SHR	HTN	SBP, DBP	NR	NR	Manip	ST36, LI11	30	21
Guo 2018 [54]	SHR, male, 190 ± 10 g	SHR	HTN, Sham-acu	SBP	10	10	Manip	ST9	30	1
Wang 2018 [55]	SHR, male, 259.17 ± 9.71	SHR	HTN	SBP, DBP	12	16	Manip + EA	ST36, LI11	15	20
Wang 2018 [56]	SHR, male, NR	SHR	HTN, Sham-acu	SBP, MAP	12	14	MA	ST36, LR3	15	14
Wang 2018 [57]	SHR, male, 225 ± 25 g	SHR	HTN	SBP	9	13	Manip	LR3	20	24
Wang 2018 [58]	SHR, male, 259.17 ± 9.71	SHR	HTN	SBP, DBP	12	16	Manip + EA	ST36, LI11	15	20
Deng 2018 [59]	SHR, male, 190 ± 10 g	SHR	HTN, Sham-acu	SBP	12	16	Manip	KI3	5	24
Huang 2018 [60]	SHR, male, 235 ± 15 g	SHR	HTN, Sham-acu	SBP, DBP	11	15	Manip	LR3, KI3	15	24
Ma 2018 [61]	SHR, male, 190 ± 10 g	SHR	HTN	SBP	9	13	Manip	ST9	20	24
Dong 2018 [62]	SHR, male, 295 ± 15	SHR	HTN	SBP, DBP	17	25	MA	ST36, ST9, LI11	20	48
Zhao 2018 [63]	SHR, male, 190 ± 10 g	SHR	HTN	SBP	10	14	Manip	ST9	20	28
Zheng 2018 [64]	SHR, male, 275 ± 25 g	SHR	HTN	SBP	17	21	Manip	ST9	20	28
Li 2018 [65]	SHR, male, 240 ± 10	SHR	HTN, Sham-acu	SBP	12	16	Manip	ST36, PC6, BL15, BL20	30	28
Luo 2018 [66]	SHR, male, 190 ± 10 g	SHR	HTN, Sham-acu	MAP	12	16	EA	ST36, DU20	20	28
Cui 2019 [67]	SHR, male, 240 ± 20 g	SHR	HTN	SBP	10-11	18-19	MA	ST36, ST7, LI11	30	40
Luo 2019 [9]	SHR, male, 230 ± 20 g	SHR	HTN, Sham-acu	SBP	12	16	Manip	LR3	5	24
Wang 2019 [68]	SHR, male, 235 ± 15 g	SHR	HTN	SBP	12	16	EA	DU20, LR3	20	30
Wang 2019 [69]	SHR, male, 280 ± 8 g	SHR	HTN, Sham-acu	SBP, DBP	13	14	Manip	LR3	10	7
Ma 2019 [70]	SHR, male, NR	SHR	HTN	SBP, MAP	12	14	MA	LR3	20	14
Ji 2019 [71]	SHR, male, 215 ± 15 g	SHR	HTN	SBP	11	15	Manip	DU20, LR3	20	28
Zheng 2019 [72]	SHR, male, 275 ± 25 g	SHR	HTN	SBP, DBP	17	21	EA	ST7	20	28
Ning 2019 [73]	SHR, male, 300 ± 30 g	SHR	HTN	SBP	16	28	EA	BL23	15	42
Sun 2019 [74]	SHR, male, 275 ± 25 g	SHR	HTN	MAP	17	21	EA	ST7	20	28
Cui 2020 [75]	SHR, male, 180 ± 20 g	SHR	HTN	SBP	10	14	MA	ST36, ST7, LI11	30	20
Hao 2020 [76]	SHR, male, 220 ± 30 g	SHR	HTN	SBP	10	12	Manip	LR3	20	14
Fang 2020 [77]	SHR, male, 200 ± 20 g	SHR	HTN	SBP	9	13	Manip	ST36, LI4, LR3	5	28
Sun 2020 [78]	SHR, male, 220 ± 30 g	SHR	HTN	SBP	10	12	Manip	LR3	20	15
Sun 2020 [79]	SHR, male, 275 ± 25 g	SHR	HTN	SBP, DBP	17	21	EA	ST7	20	28
Yang 2020 [80]	SHR, male, NR	SHR	HTN	SBP, DBP	16	20	Other	LR3	1	20

NR: not reported; SHR: spontaneously hypertension rat; HTN: hypertension; Sham-acu: sham-acupuncture; SBP: systolic blood pressure; DBP: diastolic blood pressure; MAP: mean arterial pressure; EA: electroacupuncture; MA: manual acupuncture; Manip: manipulation.

**Table 2:** Quality assessment according to the Animal Research: Reporting In Vivo Experiment (ARRIVE) guidelines.

Studies	ARRIVE items																				
	1	2	3	4	5	6	7	8	9	10	11	12	13	14	15	16	17	18	19	20	Total
Chen 2001 [15]	0	1	1	1	0	1	0	2	0	0	0	1	0	0	2	2	0	1	1	2	15
Chen 2002 [16]	1	1	1	1	0	1	0	1	0	0	0	1	0	0	2	2	0	0	1	2	14
Liu 2003 [17]	0	1	1	1	0	1	0	1	0	1	0	1	0	1	0	2	0	1	1	0	12
Su 2003 [18]	1	1	1	1	0	1	0	1	0	0	0	0	0	1	2	2	0	0	1	2	14
Yang 2006 [19]	1	1	1	1	0	1	1	2	0	1	0	1	1	1	2	2	0	1	2	0	19
Yang 2008 [20]	1	2	1	1	0	1	1	1	0	1	0	1	2	2	2	2	0	1	1	2	22
Wei 2008 [21]	1	2	0	1	0	1	1	1	1	1	0	1	0	1	2	2	1	1	1	0	18
Qian 2008 [22]	1	1	0	1	0	1	0	1	0	1	0	1	0	1	2	2	0	0	1	2	15
Liu 2009 [23]	0	1	0	1	0	1	1	1	0	0	1	0	2	1	2	2	0	1	1	2	17
Jiang 2010 [24]	1	2	2	1	2	1	1	2	1	0	1	2	2	2	2	2	2	1	1	2	30
Wang 2010 [25]	1	1	1	1	0	1	1	2	2	1	0	1	2	1	0	2	0	1	1	2	21
Xiong 2011 [26]	1	1	2	1	1	1	1	1	2	0	1	0	0	0	2	2	0	1	2	2	21
Wang 2011 [27]	1	1	1	1	1	1	1	1	1	0	0	1	1	1	2	1	0	1	1	2	19
Tian 2012 [28]	1	2	2	1	2	1	1	2	2	1	0	0	0	1	0	2	0	1	1	0	20
Duan 2013 [29]	1	1	1	1	0	1	1	2	2	1	1	1	0	1	2	2	0	1	1	0	20
Chen 2013 [30]	1	1	1	1	2	1	1	2	0	1	1	1	0	1	2	2	0	1	1	2	22
Wang 2013 [31]	1	1	0	0	0	0	2	2	2	0	0	0	0	2	2	2	0	0	1	0	15
Xu 2014 [32]	1	1	1	1	0	1	2	2	2	1	1	0	1	1	2	2	1	1	1	0	22
Yu 2014 [33]	1	2	1	1	0	1	2	2	2	1	0	1	2	2	2	2	0	1	1	0	24
Shen 2015 [34]	1	1	1	1	0	1	2	2	2	1	1	1	0	1	2	2	0	1	1	0	21
Liu 2015 [35]	0	1	1	1	0	2	2	2	0	0	1	1	2	1	1	2	0	1	2	2	22
Liu 2015 [36]	1	1	1	2	0	1	1	2	0	1	1	1	2	1	2	1	0	1	1	2	22
Zeng 2015 [37]	1	2	1	2	0	1	1	2	0	1	0	1	2	1	0	1	0	2	1	0	19
Tian 2015 [38]	1	1	1	1	0	1	1	2	2	1	1	1	1	1	2	2	1	1	1	0	22
Sun 2015 [39]	1	1	1	1	0	1	2	2	2	1	1	1	1	1	2	2	2	1	1	0	24
Zhang 2015 [40]	1	1	1	1	0	1	2	2	0	1	0	1	0	2	2	2	0	1	1	0	19
Fu 2015 [41]	1	1	2	1	0	1	1	2	2	2	0	2	2	1	2	2	0	1	1	2	26
Chang Lee 2016 [42]	1	1	2	1	1	1	2	1	2	1	1	0	0	0	2	2	0	1	1	2	22
Chen 2016 [43]	1	1	2	1	0	1	2	2	2	1	1	1	2	1	2	2	0	2	1	2	27
Tan 2016 [44]	1	1	2	0	2	1	1	1	0	0	0	0	2	0	2	2	0	1	1	2	19
Wang 2016 [45]	1	1	1	1	0	1	1	2	2	1	0	0	0	1	2	1	0	1	1	0	17
Wang 2016 [46]	1	1	1	1	0	1	2	2	2	1	0	1	2	1	2	2	0	1	1	2	24
Wang 2016 [47]	1	1	0	1	0	1	2	2	1	1	1	1	0	1	2	2	0	1	1	0	19
Xin 2016 [48]	0	1	1	1	0	0	1	2	0	0	0	1	1	0	0	1	0	1	1	0	11
Wang 2017 [49]	0	1	1	1	0	1	1	2	2	1	1	1	2	1	0	2	0	1	1	2	21
Zhang 2017 [50]	1	1	1	0	0	1	1	1	1	1	0	1	0	1	2	2	0	1	1	2	18
Guo 2017 [51]	1	1	1	1	0	1	2	2	0	1	1	1	0	0	2	2	0	1	1	2	20
Xin 2017 [52]	1	2	2	2	2	1	2	2	1	0	0	0	2	1	2	2	0	1	1	0	24
Xin 2017 [53]	1	1	0	0	0	1	1	1	1	0	0	1	0	1	2	2	0	0	0	2	14
Guo 2018 [54]	1	1	1	1	2	1	1	2	1	1	1	1	1	1	2	2	0	1	1	2	24
Wang 2018 [55]	1	1	1	1	0	1	2	2	1	0	1	1	2	2	2	2	0	1	2	2	25
Wang 2018 [56]	1	2	2	2	1	1	1	1	2	1	0	0	2	1	2	2	0	1	1	2	25
Wang 2018 [57]	1	1	2	1	0	1	1	2	1	1	1	1	0	1	2	2	0	1	1	2	22
Wang 2018 [58]	1	1	2	1	1	1	2	2	1	0	1	1	2	2	0	2	0	1	2	2	25
Deng 2018 [59]	1	1	0	1	0	1	2	2	2	1	0	0	2	2	1	2	0	1	1	2	22
Huang 2018 [60]	1	1	2	1	2	1	1	2	2	1	1	0	2	2	2	2	0	1	1	2	27
Ma 2018 [61]	1	1	1	1	0	1	1	2	0	1	1	1	0	0	2	2	0	0	1	2	18
Dong 2018 [62]	1	1	1	1	0	1	2	1	0	1	1	1	2	0	2	2	0	1	1	2	21
Zhao 2018 [63]	1	1	1	1	2	1	2	2	2	1	1	0	0	0	2	2	0	1	1	2	23
Zheng 2018 [64]	1	1	1	1	0	1	1	2	2	1	1	0	0	0	2	2	0	1	1	2	20
Li 2018 [65]	1	1	1	2	1	1	1	2	2	1	0	0	0	1	2	2	0	1	1	0	20
Luo 2018 [66]	1	1	2	1	2	1	1	2	2	1	0	0	2	2	2	2	0	1	1	2	26
Cui 2019 [67]	1	1	1	1	0	1	1	2	2	1	1	0	0	1	2	2	0	1	1	0	19
Luo 2019 [9]	0	1	2	1	2	1	2	2	2	1	1	1	2	2	2	2	0	1	1	2	28
Wang 2019 [68]	1	2	2	1	2	1	2	2	2	0	1	1	0	1	0	2	0	1	1	0	22
Wang 2019 [69]	1	1	1	1	2	1	1	2	2	1	1	2	2	0	2	2	0	2	1	2	27
Ma 2019 [70]	1	2	2	1	1	1	1	1	2	1	1	0	2	0	2	2	0	1	1	2	24
Ji 2019 [71]	1	1	2	1	2	1	1	2	0	1	1	1	0	0	2	2	0	1	1	2	22
Zheng 2019 [72]	1	1	1	1	0	1	2	2	0	1	1	1	0	1	2	2	0	1	1	2	21
Ning 2019 [73]	1	1	1	1	0	1	1	2	0	0	1	0	2	2	2	2	1	1	1	2	22
Sun 2019 [74]	1	1	1	1	0	1	1	2	0	1	1	1	2	1	0	1	0	1	1	2	19
Cui 2020 [75]	1	1	1	1	0	1	1	2	0	1	1	2	0	1	2	2	0	1	1	2	21
Hao 2020 [76]	1	1	1	1	2	1	2	2	2	1	1	1	0	1	2	2	0	1	1	2	25
Fang 2020 [77]	1	1	0	1	0	1	2	2	0	1	1	1	0	1	2	2	0	1	1	2	20
Sun 2020 [78]	1	1	2	1	2	1	1	2	0	1	1	0	0	1	2	2	0	1	1	2	22
Sun 2020 [79]	1	1	2	1	1	1	1	2	0	1	1	1	0	1	2	2	0	1	1	2	22
Yang 2020 [80]	1	1	1	1	0	1	2	1	0	1	1	0	2	0	2	2	0	1	1	2	20
Category score (quality obtained)	60	77	78	68	38	66	86	117	69	51	40	49	61	64	114	128	8	64	71	94	1403
Maximum score (quality expected)	67	134	134	134	134	134	134	134	134	134	67	134	134	134	134	134	134	134	134	134	2546
Ratio: quality score/maximum score	0.90	0.57	0.58	0.51	0.29	0.49	0.64	0.87	0.51	0.38	0.60	0.37	0.46	0.48	0.85	0.96	0.06	0.48	0.53	0.70	0.55106

1: title; 2: abstract; 3: introduction-background; 4: introduction-objectives; 5: methods-ethical statement; 6: study design; 7: experimental procedure; 8: experimental animals; 9: housing and husbandry; 10: sample size; 11: allocation; 12: experimental outcomes; 13: statistics; 14: results-baseline data; 15: number analysed; 16: outcome and estimation; 17: adverse events; 18: discussion-interpretation/scientific implications; 19: general applicability/relevance; 20: funding; Total: represents total score obtained by each study out of a maximum of 38 points.

**Table 3:** Risk of bias assessment according to the Systematic Review Centre for Laboratory Animal Experimentation (SYRCLE) tool.

Studies	SYRCLE items									
	1	2	3	4	5	6	7	8	9	10
Chen 2001 [15]	?	n	n	n	n	n	n	y	y	y
Chen 2002 [16]	?	n	n	n	n	n	n	y	y	y
Liu 2003 [17]	?	y	n	n	n	n	n	?	y	y
Su 2003 [18]	?	?	n	n	n	n	n	y	y	y
Yang 2006 [19]	?	y	n	n	n	n	n	y	y	y
Yang 2008 [20]	?	n	n	n	n	n	n	y	y	y
Wei 2008 [21]	?	?	n	n	n	n	n	y	y	y
Qian 2008 [22]	?	?	n	n	n	n	n	y	y	y
Liu 2009 [23]	y	?	n	n	n	n	n	y	y	y
Jiang 2010 [24]	?	y	n	n	n	n	n	y	y	y
Wang 2010 [25]	?	y	n	n	n	n	n	?	y	y
Xiong 2011 [26]	?	?	n	n	n	n	n	y	y	y
Wang 2011 [27]	?	y	?	n	n	n	n	y	y	y
Tian 2012 [28]	?	n	n	n	n	n	n	?	y	y
Duan 2013 [29]	y	?	n	n	n	n	n	y	y	y
Chen 2013 [30]	y	?	n	n	n	n	n	y	y	y
Wang 2013 [31]	?	?	n	n	n	n	n	y	y	y
Xu 2014 [32]	y	?	n	n	n	n	n	y	y	y
Yu 2014 [33]	?	y	n	n	n	n	n	y	y	y
Shen 2015 [34]	y	y	n	n	n	n	n	y	y	y
Liu 2015 [35]	y	y	n	n	n	n	n	n	y	y
Liu 2015 [36]	y	y	n	n	n	n	n	y	y	y
Zeng 2015 [37]	?	y	n	n	n	n	n	?	y	y
Tian 2015 [38]	y	?	n	n	n	n	n	y	y	y
Sun 2015 [39]	y	?	n	n	n	n	n	y	y	y
Zhang 2015 [40]	?	y	n	n	n	n	n	y	y	y
Fu 2015 [41]	?	y	n	n	n	n	n	y	y	y
Chang Lee 2016 [42]	?	?	n	n	n	n	n	y	y	y
Chen 2016 [43]	?	?	n	n	n	n	n	y	y	y
Tan 2016 [44]	?	n	n	n	n	n	n	y	y	y
Wang 2016 [45]	?	y	n	n	n	n	n	y	y	y
Wang 2016 [46]	?	y	n	n	n	n	n	y	y	y
Wang 2016 [47]	y	?	n	n	n	n	n	y	y	y
Xin 2016 [48]	n	y	n	n	n	n	n	n	y	y
Wang 2017 [49]	y	?	n	n	n	n	n	?	y	y
Zhang 2017 [50]	?	?	n	n	n	n	n	y	y	y
Guo 2017 [51]	y	n	n	n	n	n	n	y	y	y
Xin 2017 [52]	n	?	n	n	n	n	n	y	y	y
Xin 2017 [53]	?	y	n	n	n	n	n	y	y	y
Guo 2018 [54]	y	?	n	n	n	n	n	y	y	y
Wang 2018 [55]	y	?	n	n	n	n	n	y	y	y
Wang 2018 [56]	?	?	n	n	n	n	n	y	y	y
Wang 2018 [57]	y	y	n	n	n	n	n	y	y	y
Wang 2018 [58]	y	n	n	n	n	n	?	?	y	y
Deng 2018 [59]	?	y	n	n	n	n	n	?	y	y
Huang 2018 [60]	y	y	n	n	n	n	n	y	y	y
Ma 2018 [61]	y	n	n	n	n	n	n	y	y	y
Dong 2018 [62]	y	n	n	n	n	n	n	y	y	y
Zhao 2018 [63]	y	n	n	n	n	n	n	y	y	y
Zheng 2018 [64]	y	?	n	n	n	n	n	y	y	y
Li 2018 [65]	?	?	n	n	n	n	n	y	y	y
Luo 2018 [66]	?	y	n	n	n	n	n	y	y	y
Cui 2019 [67]	y	?	n	n	n	n	n	y	y	y
Luo 2019 [9]	y	y	n	n	n	n	n	y	y	y
Wang 2019 [68]	y	?	n	n	n	n	n	?	y	y
Wang 2019 [69]	y	?	n	n	n	n	n	y	y	y
Ma 2019 [70]	y	?	n	n	n	n	n	y	y	y
Ji 2019 [71]	y	n	n	n	n	n	n	y	y	y
Zheng 2019 [72]	y	?	n	n	n	n	n	y	y	y
Ning 2019 [73]	y	y	n	n	n	n	n	y	y	y
Sun 2019 [74]	y	y	n	n	n	n	n	?	y	y
Cui 2020 [75]	y	?	n	n	n	n	n	y	y	y
Hao 2020 [76]	y	?	n	n	n	n	n	y	y	y
Fang 2020 [77]	y	?	n	n	n	n	n	y	y	y
Sun 2020 [78]	y	?	n	n	n	n	n	y	y	y
Sun 2020 [79]	y	?	n	n	n	n	n	y	y	y
Yang 2020 [80]	y	n	n	n	n	n	n	y	y	y

1: sequence generation; 2: baseline characteristics; 3: allocation concealment; 4: random housing; 5: blinding of participants and personnel; 6: random outcome assessment; 7: blinding of outcome assessment; 8: incomplete outcome data; 9: selective outcome reporting; 10: other bias; y: low risk of bias; ?: unclear; n: high risk of bias.

## Data Availability

The data are available on request to the corresponding author.

## Supplementary Materials

Supplementary Materials Tables S1–S5: subgroup analysis. Table S6: details of Egger's test. Figures S1–S6: sensitivity analysis.
