# Supplementary material for: Acupuncture for Hypertension in Animal Models: A Systematic Review and Meta-Analysis
Source: Evid Based Complement Alternat Med. 2021 Oct 11;2021:8171636. doi: 10.1155/2021/8171636 (PMC8523269; doi:10.1155/2021/8171636)
Supplement: Supplementary Materials — Tables S1–S5: subgroup analysis. Table S6: details of Egger's test. Figures S1–S6: sensitivity analysis. [file 8171636.f1.zip › Supplementary Materials Figure Legends.docx]

Figure S1. Sensitivity analysis of SBP: acupuncture vs hypertension.

Figure S2. Sensitivity analysis of SBP: acupuncture vs sham-acupuncture.

Figure S3. Sensitivity analysis of DBP: acupuncture vs hypertension.

Figure S4. Sensitivity analysis of DBP: acupuncture vs sham-acupuncture.

Figure S5. Sensitivity analysis of MAP: acupuncture vs hypertension.

Figure S6. Sensitivity analysis of MAP: acupuncture vs sham-acupuncture.
