# Supplementary material for: Acupuncture for Hypertension in Animal Models: A Systematic Review and Meta-Analysis
Source: Evid Based Complement Alternat Med. 2021 Oct 11;2021:8171636. doi: 10.1155/2021/8171636 (PMC8523269; doi:10.1155/2021/8171636)
Supplement: Supplementary Materials — Tables S1–S5: subgroup analysis. Table S6: details of Egger's test. Figures S1–S6: sensitivity analysis. [file 8171636.f1.zip › Table S1.docx]

Table S1. Subgroup analysis of acupuncture for SBP between acupuncture and hypertension.

| **Subgroup variables** | **No. of studies** | **Pooled WMD (95%CI)** | **Measure of heterogeneity** | | | **Weight (%)** |
| --- | --- | --- | --- | --- | --- | --- |
|  |  |  | χ2 | P | I^2^ |  |
| **Treatment** |  | | | | | |
| MA | 5 | -32.80 (-47.05, -18.54) | 224.70 | ＜0.001 | 98.2% | 8.77 |
| Manip | 33 | -24.27 (-29.56, -18.98) | 1672.62 | ＜0.001 | 98.1% | 54.58 |
| EA | 16 | -23.74 (-26.06, -21.42) | 30.78 | 0.009 | 51.3% | 26.61 |
| EA+Manip | 4 | -30.06 (-45.55, -14.56) | 128.97 | ＜0.001 | 97.7% | 6.96 |
| Other | 2 | -18.72 (-26.56, -10.88) | 0.05 | 0.82 | 0.0% | 3.09 |
| **Age for acupuncture** |  | | | | | |
| 1-10 weeks | 12 | -15.54 (-20.11, -10.98) | 315.56 | ˂0.001 | 96.5% | 20.88 |
| 11-20weeks | 35 | -26.91 (-31.97, -21.86) | 930.56 | ˂0.001 | 96.3% | 57.52 |
| 21-34 weeks | 4 | -31.77 (-48.03, -15.51) | 83.33 | ˂0.001 | 96.4% | 6.56 |
| NR | 9 | -29.83 (-40.97, -18.69) | 199.59 | ˂0.001 | 96.0% | 15.04 |
| **Age for BP measurement** |  | | | | | |
| 10-20weeks | 36 | -24.07 (-29.09, -19.06) | 2029.19 | ˂0.001 | 98.3% | 61.45 |
| 21-37weeks | 15 | -25.77 (-30.64, -20.90) | 109.32 | ˂0.001 | 87.2% | 23.51 |
| NR | 9 | -29.83 (-40.97, -18.69) | 199.59 | ˂0.001 | 96.0% | 15.04 |
| **Duration** |  | | | | | |
| Less than 5 minutes | 7 | -23.24 (-32.14, -14.33) | 20.38 | 0.002 | 70.6% | 9.78 |
| 5-10 minutes | 14 | -22.39 (-29.29, -15.50) | 196.91 | ˂0.001 | 93.4% | 23.57 |
| 11-20 minutes | 27 | -24.76 (-30.32, -19.21) | 1487.28 | ˂0.001 | 98.3% | 46.09 |
| 30 minutes | 11 | -32.15 (-42.20, -22.10) | 642.37 | ˂0.001 | 98.4% | 18.89 |
| NR | 1 | -17.50 (-25.94, -9.06) | 0 | .. | 0 | 1.66 |
| **Sessions** |  | | | | | |
| 1 time | 2 | -22.33 (-49.33, 4.66) | 19.10 | ˂0.001 | 94.8% | 3.33 |
| 2-10 times | 8 | -19.86 (-26.92, -12.79) | 40.40 | ˂0.001 | 82.7% | 13.21 |
| 11-20 times | 18 | -28.47 (-36.27, -20.67) | 1148.99 | ˂0.001 | 98.5% | 29.44 |
| 21-30 times | 22 | -26.35 (-33.40, -19.30) | 1159.10 | ˂0.001 | 98.2% | 37.51 |
| More than 40 times | 10 | -22.10 (-25.26, -18.94) | 20.62 | 0.014 | 56.4% | 16.51 |
| **Frequency** |  | | | | | |
| 1 | 2 | -22.33 (-49.33, 4.66) | 19.10 | ˂0.0001 | 94.8% | 3.33 |
| Qod | 2 | -27.63 (-33.67, -21.58) | 0.46 | 0.50 | 0.0% | 3.20 |
| 5d/w | 11 | -25.80 (-33.19, -18.41) | 106.02 | ˂0.0001 | 90.6% | 16.84 |
| 6d/w | 11 | -22.23(-29.76, -14.70) | 410.98 | ˂0.0001 | 97.6% | 19.3 |
| 7d/w | 33 | -25.41(-30.87, -19.94) | 1627.48 | ˂0.0001 | 98.0% | 55.61 |
| Other | 1 | -61.68(-67.99, -55.37) | 0.00 | .. | 0 | 1.72 |

Note NR: not reported; WMD: weighted mean difference; HTN: hypertension; SBP: systolic blood pressure; DBP: diastolic blood pressure; MAP: mean arterial pressure; EA: electroacupuncture; MA: manual acupuncture; Manip: manipulation; Qod: 1 time every 2 days.
