# Supplementary material for: Acupuncture for Hypertension in Animal Models: A Systematic Review and Meta-Analysis
Source: Evid Based Complement Alternat Med. 2021 Oct 11;2021:8171636. doi: 10.1155/2021/8171636 (PMC8523269; doi:10.1155/2021/8171636)
Supplement: Supplementary Materials — Tables S1–S5: subgroup analysis. Table S6: details of Egger's test. Figures S1–S6: sensitivity analysis. [file 8171636.f1.zip › Table S2.docx]

Table S2. Subgroup analysis of acupuncture for SBP between acupuncture and sham-acupuncture.

| **Subgroup variables** | **No. of studies** | **Pooled WMD (95%CI)** | **Measure of heterogeneity** | | | **Weight (%)** |
| --- | --- | --- | --- | --- | --- | --- |
|  |  |  | **χ2** | **P** | **I^2^** |  |
| **Treatment** |  | | | | | |
| MA | 1 | -33.95 (-40.65, -27.25) | 0.00 | .. | 0.0% | 5.63 |
| Manip | 15 | -21.19 (-28.08, -14.29) | 297.33 | ˂0.0001 | 95.3% | 78.64 |
| EA | 3 | -17.37 (-23.64, -11.10) | 5.82 | 0.06 | 65.6% | 15.73 |
| Ager for acupuncture |  | | | | | |
| 1-10 weeks | 3 | -8.86 (-11.21, -6.51) | 0.38 | 0.83 | 0.0% | 15.87 |
| 11-20 weeks | 13 | -24.61 (-30.86, -18.37) | 198.91 | ˂0.0001 | 94.0% | 69.71 |
| NR | 3 | -19.95 (-24.94, -14.97) | 1.15 | 0.56 | 0.0% | 14.42 |
| **Age for BP Measurement** |  | | | | | |
| 10-20 weeks | 14 | -22.45 (-29.52, -15.37) | 306.39 | ˂0.0001 | 95.8% | 75.44 |
| 21-37 weeks | 2 | -15.76 (-23.96, -7.57) | 1.60 | 0.207 | 37.3% | 10.15 |
| NR | 3 | -19.95 (-24.94, -14.97) | 1.15 | 0.56 | 0.0% | 14.42 |
| **Duration** |  | | | | | |
| Less than 5 minutes | 3 | -25.87 (-42.22, -9.52) | 11.75 | 0.003 | 83.0% | 14.27 |
| 5-10 minutes | 9 | -18.55 (-26.02, -11.09) | 49.25 | ˂0.0001 | 83.8% | 46.25 |
| 11-20 minutes | 2 | -36.80(-39.45, -34.16) | 0.82 | 0.36 | 0.0% | 11.67 |
| 30 minutes | 5 | -18.22 (-26.67, -9.78) | 99.24 | ˂0.0001 | 96.0% | 27.81 |
| **Sessions** |  | | | | | |
| 1 time | 2 | -12.27 (-23.51, -1.03) | 2.17 | 0.14 | 53.8% | 9.99 |
| 2-10 times | 7 | -16.75 (-24.37, -9.12) | 23.86 | ˂0.001 | 74.9% | 35.08 |
| 21-30 times | 7 | -28.45 (-34.69, -22.21) | 52.90 | ˂0.0001 | 88.7% | 37.47 |
| More than 40 times | 1 | -13.64 (-16.40, -10.88) | 0 | .. | 0 | 6.04 |
| **Frequency** |  | | | | | |
| 1 | 2 | -12.27 (-23.51, -1.03) | 2.17 | 0.141 | 53.8% | 9.99 |
| 6d/w | 4 | -25.31 (-37.39, -13.23) | 66.98 | ˂0.0001 | 95.5% | 22.99 |
| 7d/w | 13 | -21.30 (-26.71, -15.88) | 96.03 | ˂0.0001 | 87.5% | 67.02 |

Note NR: not reported; WMD: weighted mean difference; HTN: hypertension; SBP: systolic blood pressure; DBP: diastolic blood pressure; MAP: mean arterial pressure; EA: electroacupuncture; MA: manual acupuncture; Manip: manipulation.
