# Supplementary material for: Acupuncture for Hypertension in Animal Models: A Systematic Review and Meta-Analysis
Source: Evid Based Complement Alternat Med. 2021 Oct 11;2021:8171636. doi: 10.1155/2021/8171636 (PMC8523269; doi:10.1155/2021/8171636)
Supplement: Supplementary Materials — Tables S1–S5: subgroup analysis. Table S6: details of Egger's test. Figures S1–S6: sensitivity analysis. [file 8171636.f1.zip › Table S3.docx]

**Table S3. Subgroup analysis of acupuncture for DBP between acupuncture and hypertension.**

| **Subgroup variables** | **No. of studies** | **Pooled WMD (95%CI)** | **Measure of heterogeneity** | | | **Weight (%)** |
| --- | --- | --- | --- | --- | --- | --- |
|  |  |  | **χ2** | **P** | **I^2^** |  |
| **Treatment** |  | | | | | |
| MA | 1 | -14.84 (-24.41, -5.27) | 0 | .. | 0 | 4.45 |
| Manip | 11 | -24.31 (-30.19, -18.43) | 87.49 | ˂0.0001 | 88.6% | 48.74 |
| EA | 6 | -13.04 (-18.14, -7.94) | 24.46 | ˂0.0001 | 79.6% | 28.79 |
| Manip+EA | 3 | -33.81 (-38.36, -29.25) | 4.11 | 0.128 | 51.4% | 14.35 |
| Other | 1 | -5.00 (-19.47, 9.47) | 0 | .. | 0 | 3.67 |
| **Age for acupuncture** |  | | | | | |
| 1-10 weeks | 1 | -9.40 (-16.15, -2.65) | 0 | .. | 0 | 4.86 |
| 11-20 weeks | 17 | -21.98 (-27.63, -16.33) | 370.97 | <0.0001 | 95.7% | 76.93 |
| NR | 4 | -21.28 (-35.53, -7.03) | 35.67 | <0.0001 | 91.6% | 18.21 |
| **Age for BP Measurement** |  | | | | | |
| 10-20 weeks | 12 | -24.42 (-30.80, -18.03) | 234.96 | <0.0001 | 95.3% | 55.11 |
| 21-37 weeks | 6 | -14.01 (-18.65, -9.37) | 15.78 | 0.007 | 68.3% | 26.68 |
| NR | 4 | -21.28 (-35.53, -7.03) | 35.67 | <0.0001 | 91.6% | 18.21 |
| **Duration** |  | | | | | |
| Less than 5 minutes | 4 | -21.01 (-39.42, -2.60) | 17.72 | <0.001 | 83.1% | 14.20 |
| 5-10 minutes | 4 | -20.48 (-32.16, -8.79) | 46.26 | <0.001 | 93.5% | 19.83 |
| 11-20 minutes | 8 | -22.39 (-31.11, -13.66) | 310.04 | <0.0001 | 97.7% | 37.85 |
| 30 minutes | 6 | -20.37 (-28.58, -12.16) | 40.13 | <0.0001 | 87.5% | 28.13 |
| **Sessions** |  | | | | | |
| 1 time | 1 | -24.05 (-36.71, -11.39) | 0 | .. | 0 | 3.96 |
| 2-10 times | 2 | -23.71 (-51.67, 4.25) | 38.50 | <0.0001 | 97.4% | 9.82 |
| 11-20 times | 7 | -26.77 (-33.30, -20.23) | 27.92 | <0.0001 | 78.5% | 29.47 |
| 21-30 times | 9 | -18.25 (-23.69, -12.80) | 83.67 | <0.0001 | 90.4% | 42.37 |
| more than 40 times | 3 | -18.24 (-21.86, -14.62) | 0.85 | 0.655 | 0.0% | 14.37 |
| **Frequency** |  | | | | | |
| 1 | 1 | -24.05 (-36.71, -11.39) | 0 | .. | 0 | 3.96 |
| 5d/w | 6 | -25.48 (-33.47, -17.50) | 27.39 | <0.0001 | 81.7% | 24.77 |
| 6d/w | 4 | -17.50 (-19.37, -15.62) | 0.88 | 0.830 | 0.0% | 19.75 |
| 7d/w | 10 | -20.13 (-27.31, -12.94) | 121.71 | <0.0001 | 92.6% | 46.50 |
| Other | 1 | -30.85 (-36.30, -25.40) | 0 | .. | 0.0% | 5.02 |

Note NR: not reported; WMD: weighted mean difference; HTN: hypertension; SBP: systolic blood pressure; DBP: diastolic blood pressure; MAP: mean arterial pressure; EA: electroacupuncture; MA: manual acupuncture; Manip: manipulation.
