# Supplementary material for: Acupuncture for Hypertension in Animal Models: A Systematic Review and Meta-Analysis
Source: Evid Based Complement Alternat Med. 2021 Oct 11;2021:8171636. doi: 10.1155/2021/8171636 (PMC8523269; doi:10.1155/2021/8171636)
Supplement: Supplementary Materials — Tables S1–S5: subgroup analysis. Table S6: details of Egger's test. Figures S1–S6: sensitivity analysis. [file 8171636.f1.zip › Table S4.docx]

**Table S4. Subgroup analysis of acupuncture for DBP between acupuncture and sham-acupuncture.**

| **Subgroup variables** | **No. of studies** | **Pooled WMD (95%CI)** | **Measure of heterogeneity** | | | **Weight (%)** |
| --- | --- | --- | --- | --- | --- | --- |
|  |  |  | **χ2** | **P** | **I^2^** |  |
| **Treatment** |  | | | | | |
| Manip | 5 | -23.46 (-33.17, -13.76) | 38.65 | <0.0001 | 89.7% | 74.05 |
| EA | 2 | -5.13 (-16.93, 6.67) | 1.62 | 0.203 | 38.3% | 25.95 |
| **Age for acupuncture** |  | | | | | |
| 1-10 weeks | 1 | -8.90 (-18.34, 0.54) | 0 | .. | 0.0 | 15.22 |
| 11-20 weeks | 4 | -27.31 (-38.71, -15.90) | 34.05 | <0.001 | 91.2% | 58.83 |
| NR | 2 | -5.13 (-16.93, 6.67) | 1.62 | 0.203 | 38.3% | 25.95 |
| **Age for BP measurement** |  | | | | | |
| 10-20 weeks | 4 | -22.85 (-33.78, -11.92) | 37.58 | <0.001 | 92.0% | 62.19 |
| 21-37 weeks | 1 | -27.00 (-42.25, -11.76) | 0.00 | .. | 0% | 11.86 |
| NR | 2 | -5.13 (-16.93, 6.67) | 1.62 | 0.203 | 38.3% | 25.95 |
| **Duration** |  | | | | | |
| Less than 5 minutes | 2 | -31.34 (-42.40, -20.29) | 0.66 | 0.417 | 0.0% | 23.27 |
| 5-10 minutes | 2 | -21.10 (-44.36, 2.15) | 18.43 | <0.001 | 94.6% | 32.50 |
| 11-20 minutes | 1 | -16.95 (-19.05, -14.85) | 0 | .. | 0 | 18.27 |
| 30 minutes | 2 | -5.13 (-16.93, 6.67) | 1.62 | 0.203 | 38.3% | 25.95 |
| **Sessions** |  | | | | | |
| 1 time | 1 | -14.96 (-34.36, 4.44) | 0 | .. | 0 | 9.71 |
| 2-10 times | 2 | -21.10 (-44.36, 2.15) | 18.43 | <0.001 | 94.6% | 32.50 |
| 21-30 times | 4 | -18.46 (-30.06, -6.87) | 23.01 | <0.001 | 87.0% | 57.79 |
| **Frequency** |  | | | | | |
| 1 | 1 | -14.96 (-34.36, 4.44) | 0 | .. | 0 | 9.71 |
| 6d/w | 1 | -16.95 (-19.05, -14.85) | 0 | .. | 0 | 18.27 |
| 7d/w | 5 | -20.71 (-35.97, -5.44) | 54.41 | <0.001 | 92.6% | 72.02 |

Note NR: not reported; WMD: weighted mean difference; HTN: hypertension; SBP: systolic blood pressure; DBP: diastolic blood pressure; MAP: mean arterial pressure; EA: electroacupuncture; MA: manual acupuncture; Manip: manipulation.
