# Supplementary material for: Acupuncture for Hypertension in Animal Models: A Systematic Review and Meta-Analysis
Source: Evid Based Complement Alternat Med. 2021 Oct 11;2021:8171636. doi: 10.1155/2021/8171636 (PMC8523269; doi:10.1155/2021/8171636)
Supplement: Supplementary Materials — Tables S1–S5: subgroup analysis. Table S6: details of Egger's test. Figures S1–S6: sensitivity analysis. [file 8171636.f1.zip › Table S5.docx]

**Table S5. Subgroup analysis of acupuncture for MAP between acupuncture and hypertension.**

| **Subgroup variables** | **No.of studies** | **Pooled WMD (95%CI)** | **Measure of heterogeneity** | | | **Weight(%)** |
| --- | --- | --- | --- | --- | --- | --- |
|  |  |  | **χ2** | **P** | **I^2^** |  |
| **Treatment** |  | | | | | |
| MA | 2 | -36.88 (-38.82, -34.94) | 0.30 | 0.582 | 0.0% | 18.57 |
| Manip | 2 | -16.51 (-25.64, -7.38) | 8.62 | 0.003 | 88.4% | 18.20 |
| EA | 8 | -18.06 (-23.04, -13.07) | 22.32 | 0.002 | 68.6% | 63.23 |
| **Age for acupuncture** |  | | | | | |
| 1-10 weeks | 2 | -27.69 (-35.09, -20.30) | 0.01 | 0.934 | 0.0% | 13.21 |
| 11-20 weeks | 8 | -23.56 (-32.41, -14.71) | 240.65 | <0.001 | 97.1% | 69.36 |
| NR | 2 | -11.00 (-20.56, -1.45) | 4.02 | 0.045 | 75.1% | 17.43 |
| **Age for BP measurement** |  | | | | | |
| 10-20 weeks | 8 | -26.22 (-35.46, -16.99) | 216.52 | <0.001 | 96.8% | 64.78 |
| 21-37 weeks | 2 | -17.24 (-22.44, -12.04) | 1.63 | 0.202 | 38.7% | 17.78 |
| NR | 2 | -11.00 (-20.56, -1.45) | 4.02 | 0.045 | 75.1% | 17.43 |
| **Duration** |  | | | | | |
| Less than 5 minutes | 1 | -12.16 (-14.97, -9.36) | 0 | .. | 0 | 9.28 |
| 5-10 minutes | 1 | -21.50 (-27.07, -15.93) | 0 | .. | 0 | 8.92 |
| 11-20 minutes | 7 | -27.12 (-34.81, -19.43) | 78.01 | <0.001 | 92.3% | 55.33 |
| 30 minutes | 3 | -14.99 (-23.32, -6.65) | 11.06 | 0.004 | 81.9% | 26.47 |
| **Sessions** |  |  |  |  |  |  |
| 11-20 times | 6 | -26.41 (-38.20, -14.62) | 214.50 | <0.001 | 97.7% | 47.24 |
| 21-30 times | 4 | -17.32 (-25.73, -8.90) | 20.90 | <0.001 | 85.6% | 35.05 |
| more than 40 times | 2 | -19.30 (-23.50, -15.11) | 0.07 | 0.794 | 0 | 17.71 |
| **Frequency** |  | | | | | |
| 5d/w | 1 | -29.00 (-46.95, -11.05) | 0 | .. | 0.0 | 6.02 |
| 6d/w | 2 | -16.51 (-25.64, -7.38) | 8.62 | 0.003 | 88.4% | 18.20 |
| 7d/w | 7 | -21.55 (-30.61, -12.49) | 166.69 | <0.001 | 96.4% | 62.57 |
| Other | 1 | -28.66 (-52.63, -4.69) | 0 | .. | 0 | 4.70 |

Note NR: not reported; WMD: weighted mean difference; HTN: hypertension; SBP: systolic blood pressure; DBP: diastolic blood pressure; MAP: mean arterial pressure; EA: electroacupuncture; MA: manual acupuncture; Manip: manipulation; Qod: 1 time every 2 days.
