# Supplementary figures and images for: Acupuncture for Hypertension in Animal Models: A Systematic Review and Meta-Analysis
Source: Evid Based Complement Alternat Med. 2021 Oct 11;2021:8171636. doi: 10.1155/2021/8171636 (PMC8523269; doi:10.1155/2021/8171636)

# Meta-analysis random-effects estimates (linear form)

Study omitted

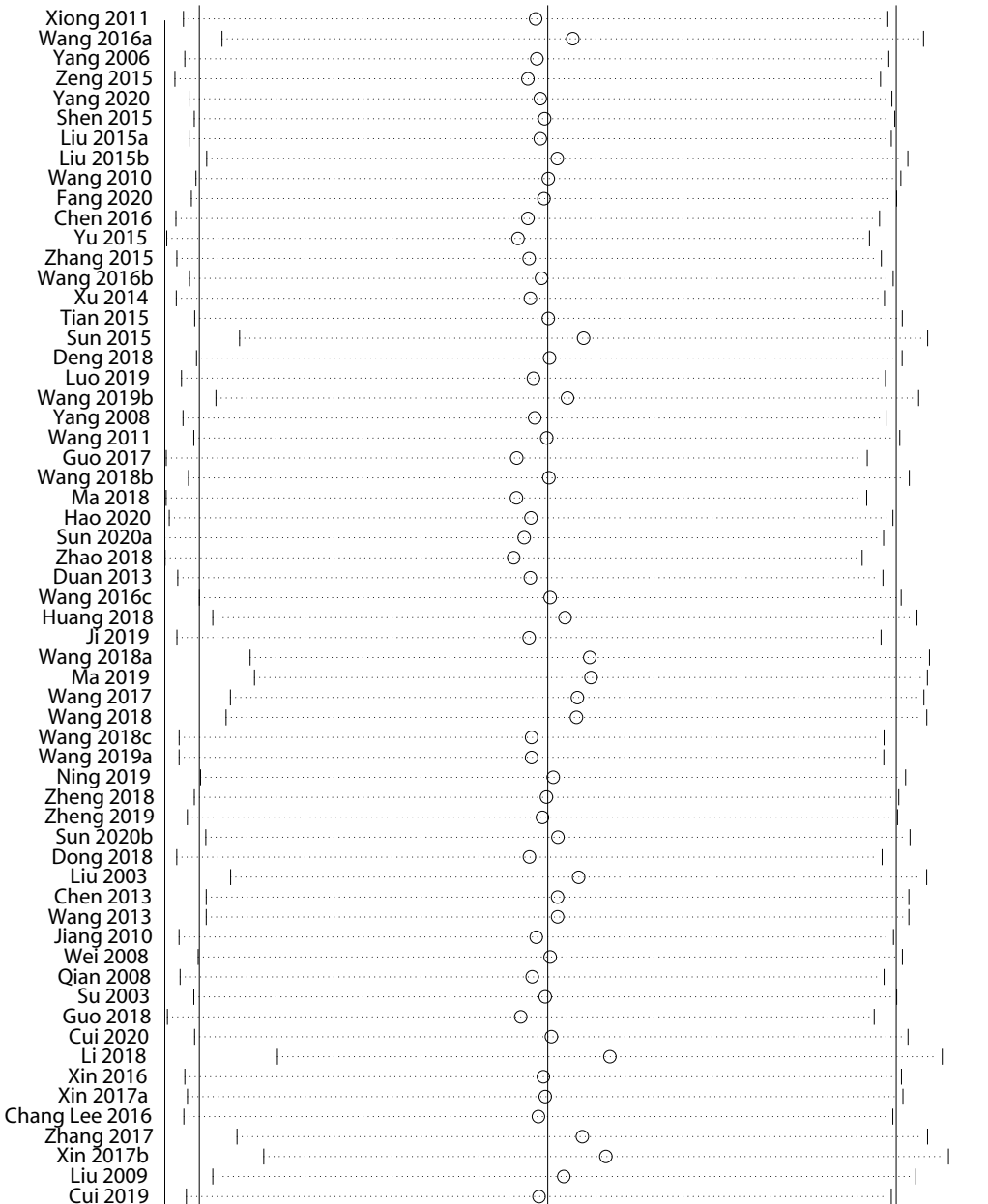

Supplement: Supplementary Materials — Tables S1–S5: subgroup analysis. Table S6: details of Egger's test. Figures S1–S6: sensitivity analysis. [file 8171636.f1.zip › Figure S1.pdf]

# Meta-analysis random-effects estimates (linear form)

Study ommited

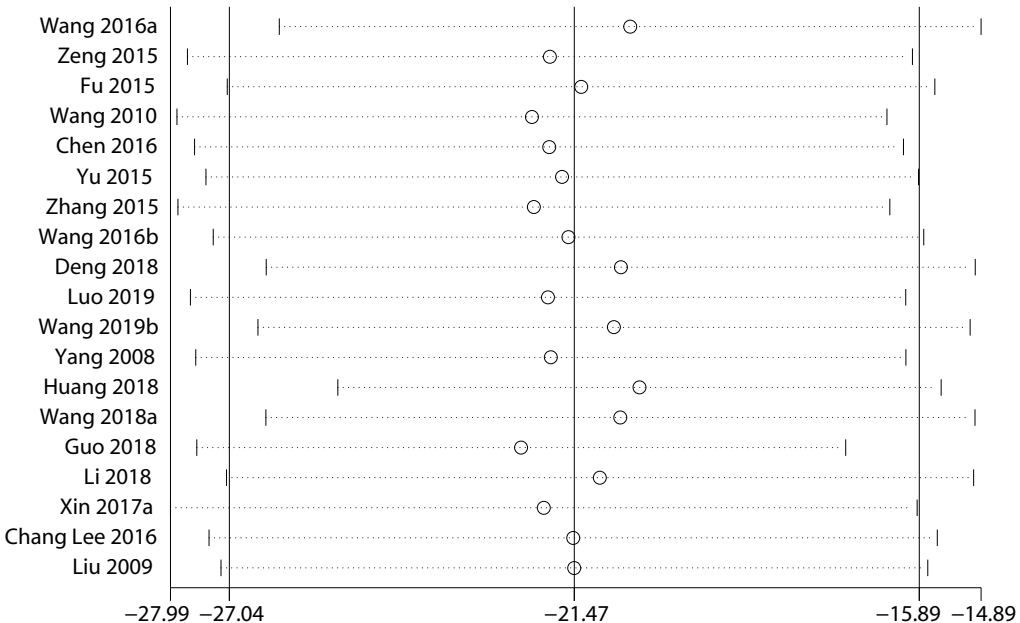

Supplement: Supplementary Materials — Tables S1–S5: subgroup analysis. Table S6: details of Egger's test. Figures S1–S6: sensitivity analysis. [file 8171636.f1.zip › Figure S2.pdf]

# Meta-analysis random-effects estimates (linear form)

Study omitted

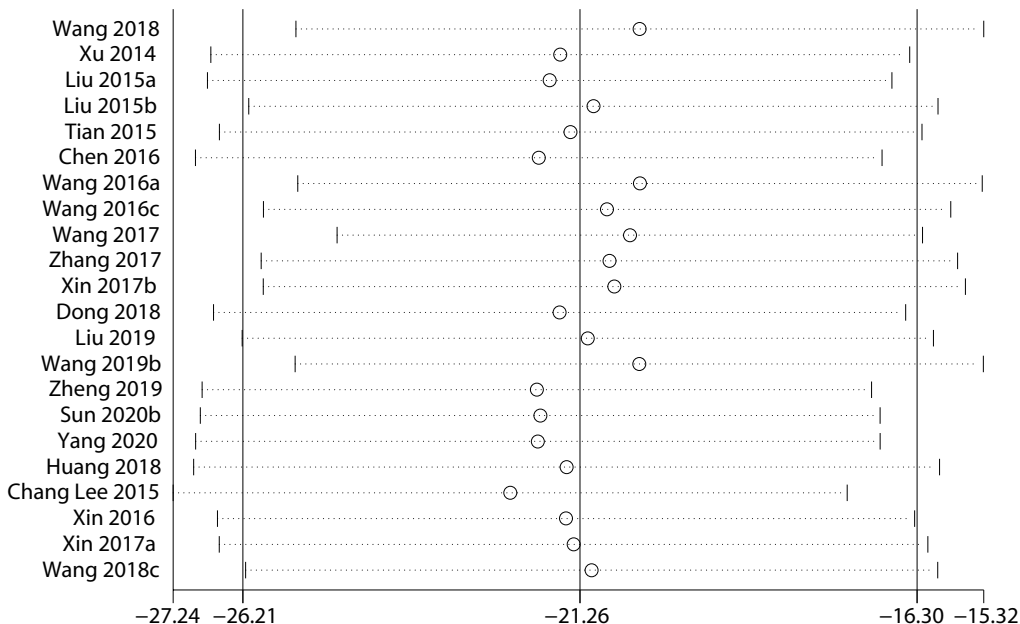

Supplement: Supplementary Materials — Tables S1–S5: subgroup analysis. Table S6: details of Egger's test. Figures S1–S6: sensitivity analysis. [file 8171636.f1.zip › Figure S3.pdf]

# Meta-analysis random-effects estimates (linear form)

Study ommited

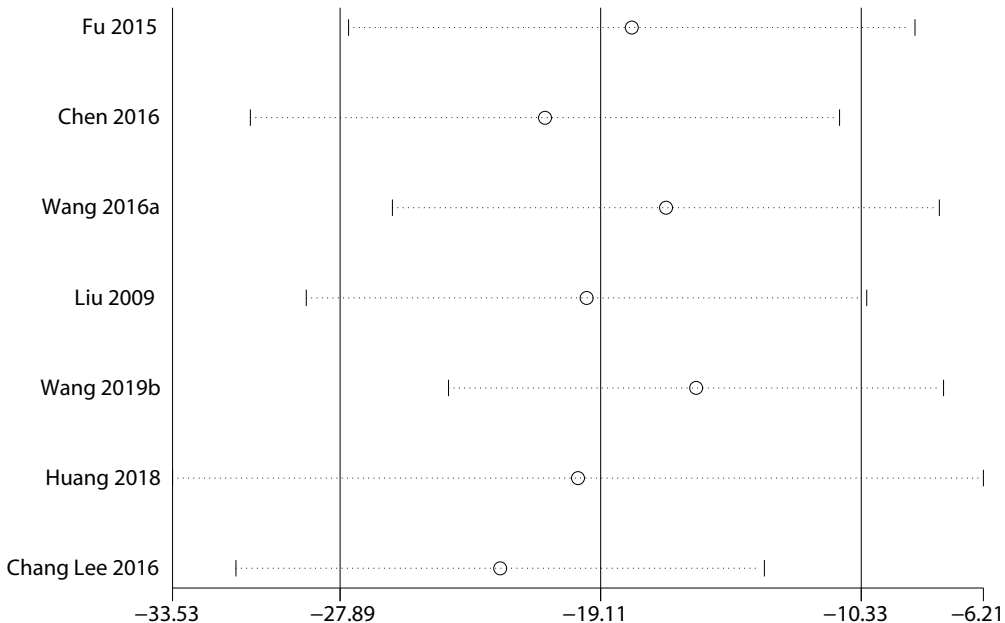

Supplement: Supplementary Materials — Tables S1–S5: subgroup analysis. Table S6: details of Egger's test. Figures S1–S6: sensitivity analysis. [file 8171636.f1.zip › Figure S4.pdf]

# Meta-analysis random-effects estimates (linear form)

Study omitted

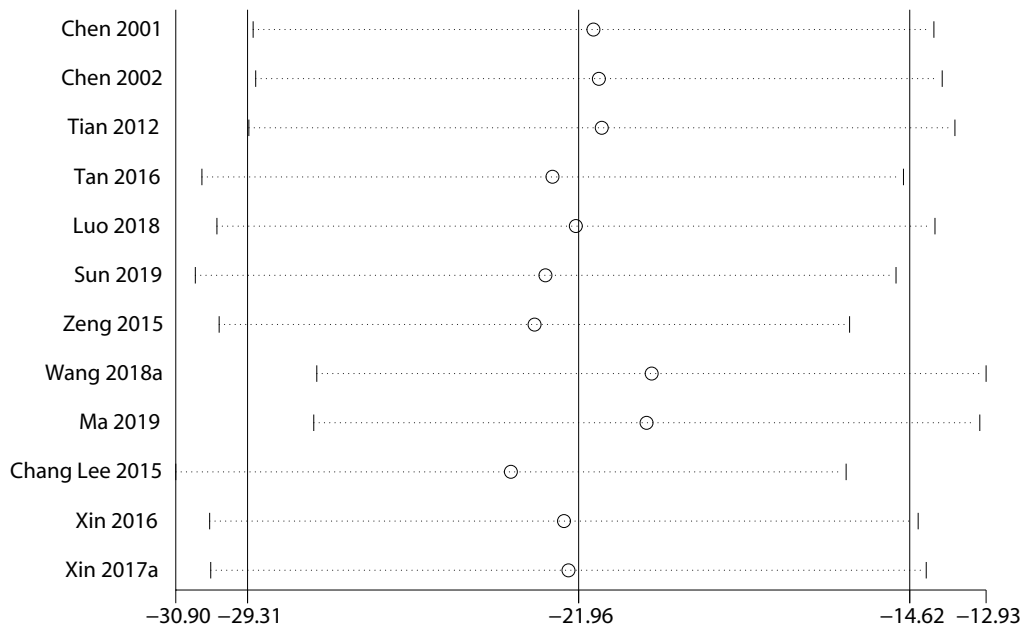

Supplement: Supplementary Materials — Tables S1–S5: subgroup analysis. Table S6: details of Egger's test. Figures S1–S6: sensitivity analysis. [file 8171636.f1.zip › Figure S5.pdf]

# Meta-analysis random-effects estimates (linear form)

Study omitted

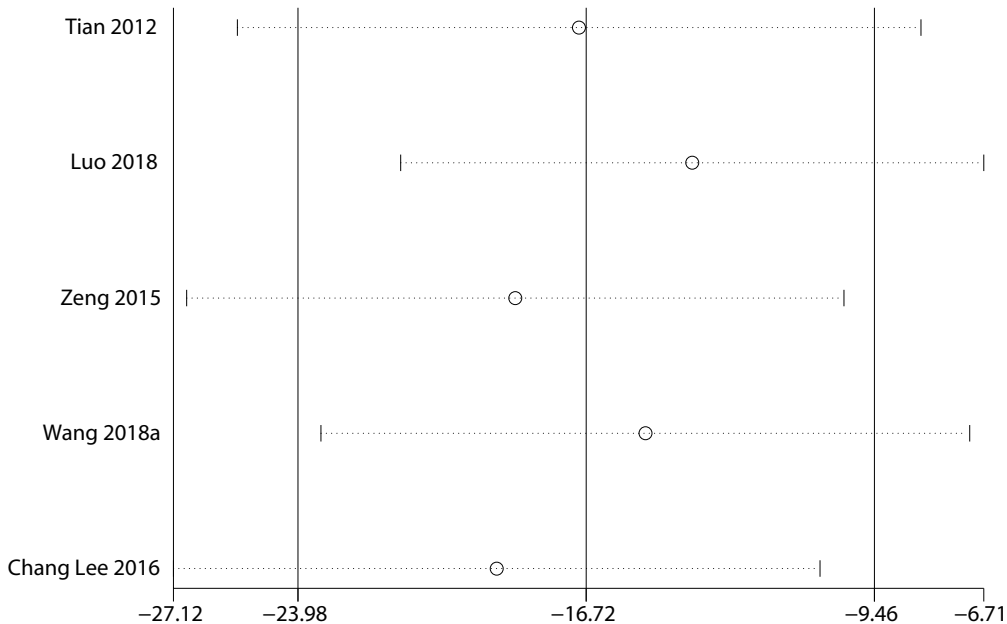

Supplement: Supplementary Materials — Tables S1–S5: subgroup analysis. Table S6: details of Egger's test. Figures S1–S6: sensitivity analysis. [file 8171636.f1.zip › Figure S6.pdf]
